# Supplementary figures and images for: Late-life dietary folate restriction reduces biosynthesis without compromising healthspan in mice
Source: Life Sci Alliance. 2024 Jul 23;7(10):e202402868. doi: 10.26508/lsa.202402868 (PMC11266815; doi:10.26508/lsa.202402868)

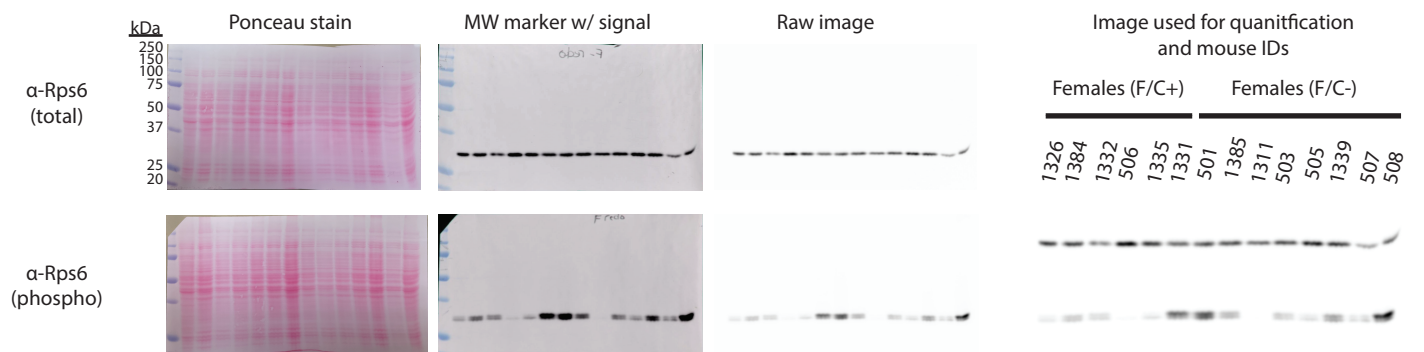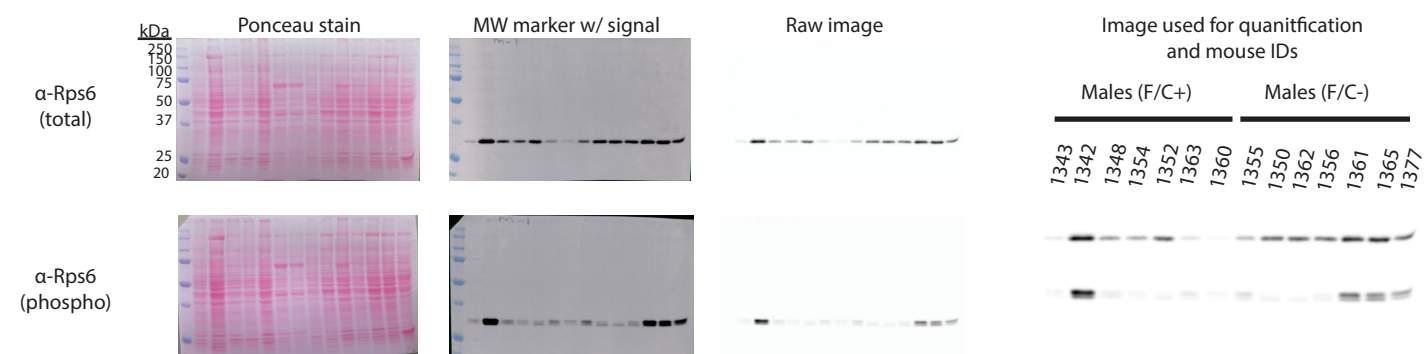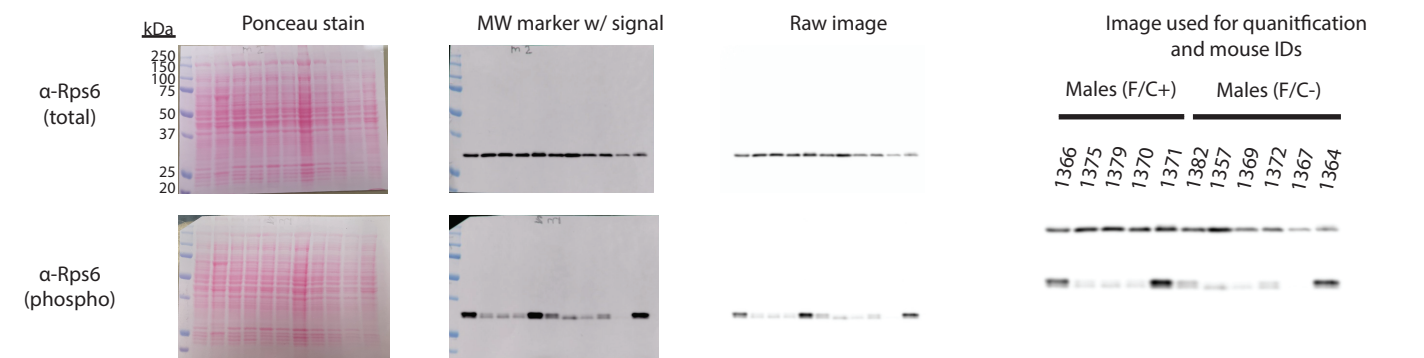

Supplement: Supplementary file 18 [file LSA-2024-02868_SdataFS13.1.pdf]

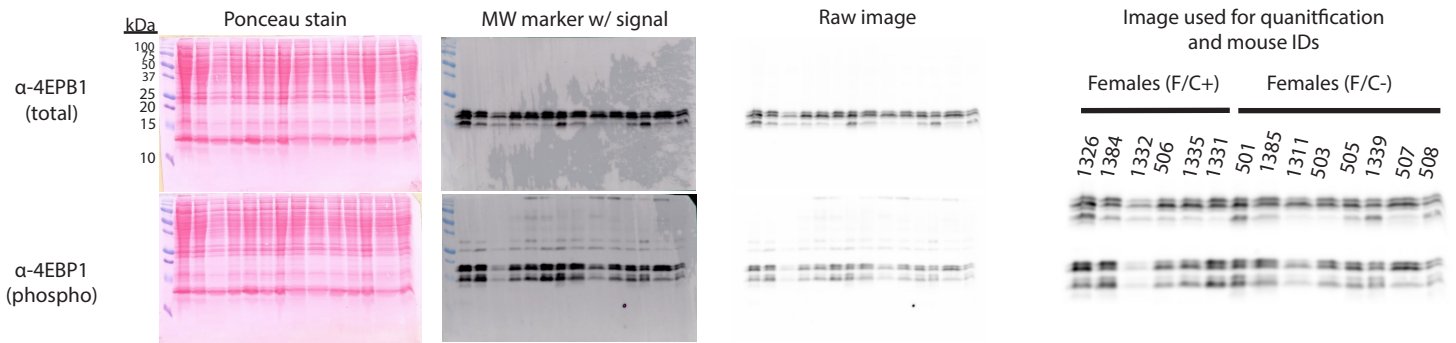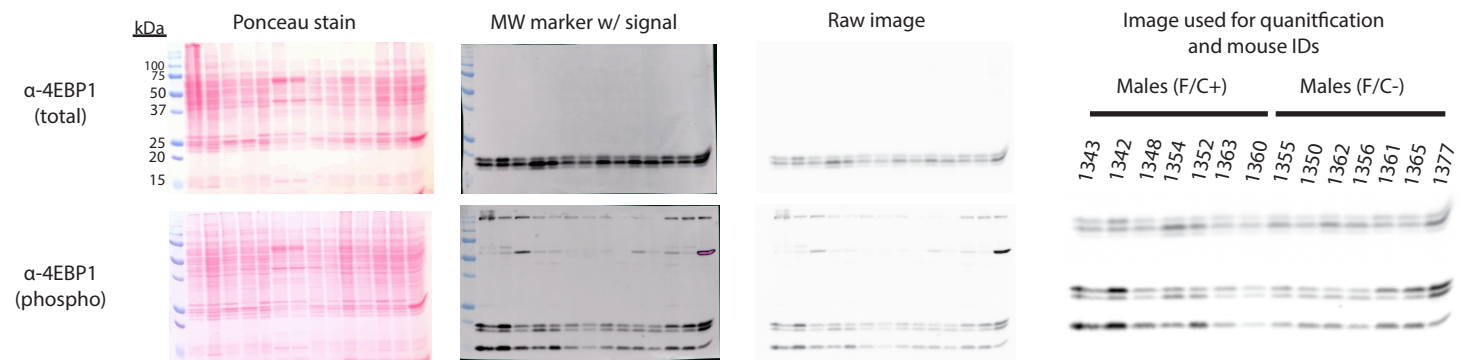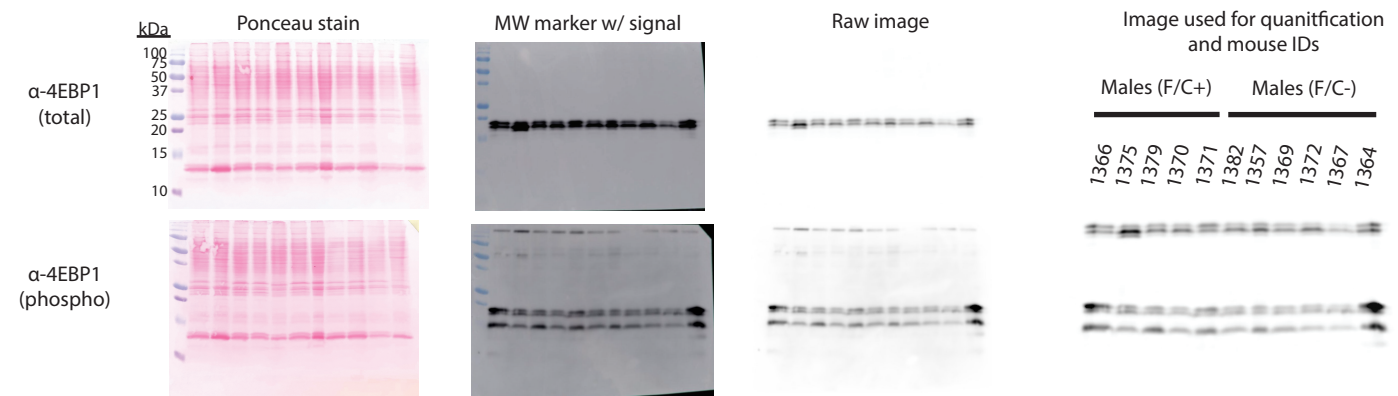

Supplement: Supplementary file 20 [file LSA-2024-02868_SdataFS14.1.pdf]
